# Web of Science use in published research and review papers 1997–2017: a selective, dynamic, cross-domain, content-based analysis

**DOI:** 10.1007/s11192-017-2622-5

**Published:** 2017-12-18

**Authors:** Kai Li, Jason Rollins, Erjia Yan

**Affiliations:** 1 Drexel University, 30N 33rd St., Philadelphia, PA 19104 USA; 2 Clarivate Analytics, 50 California St., San Francisco, CA 94111 USA

**Keywords:** Web of Science, Scientometrics, Natural language processing, Eugene Garfield

## Abstract

Clarivate Analytics’s Web of Science (WoS) is the world’s leading scientific citation search and analytical information platform. It is used as both a research tool supporting a broad array of scientific tasks across diverse knowledge domains as well as a dataset for large-scale data-intensive studies. WoS has been used in thousands of published academic studies over the past 20 years. It is also the most enduring commercial legacy of Eugene Garfield. Despite the central position WoS holds in contemporary research, the quantitative impact of WoS has not been previously examined by rigorous scientific studies. To better understand how this key piece of Eugene Garfield’s heritage has contributed to science, we investigated the ways in which WoS (and associated products and features) is mentioned in a sample of 19,478 English-language research and review papers published between 1997 and 2017, as indexed in WoS databases. We offered descriptive analyses of the distribution of the papers across countries, institutions and knowledge domains. We also used natural language processingtechniques to identify the verbs and nouns in the abstracts of these papers that are grammatically connected to WoS-related phrases. This is the first study to empirically investigate the documentation of the use of the WoS platform in published academic papers in both scientometric and linguistic terms.

## Introduction

During his long and impactful career, Eugene Garfield made significant contributions to the field of information science and scientometrics. His work has resulted in many accolades including being considered “one of the most visionary figures in information science and scientometrics” (van Raan and Wouters 2017, para. 1) and “the grandfather of Google…” (Rumsey 2010, para. 6). Garfield’s most far-reaching contributions might be the more than 1500 papers he published^1^, which are the topic of many recent retrospectives and tributes. These include Chen’s work (2017) examining the scientific impacts of Garfield’s oeuvre and all the publications that cite his works as well as Bornmann et al.’s study (2017) analyzing the historical roots of Eugene Garfield’s papers using the reference publication year spectroscopy (RPYS) method.

Perhaps an equally substantial contribution is the work Garfield did to develop the Science Citation Index (SCI) that is now part of the Web of Science (WoS) database. Influenced by Frank Shepard’s efforts to trace the connections between citing and cited legal documents in the 1870s (Adair 1955), Garfield proposed the idea of a unified index to scientific documents in his seminal paper titled “Citation Indexes for Science A New Dimension in Documentation through Association of Ideas” (Garfield 1955). Garfield defined this new concept as a “thought” index, which is an extension of a subject index by offering a more thorough coverage of the content of scientific publications; moreover, rather than relying upon a limited number of professional indexers, this new index would be built on the efforts conducted by the researchers themselves, a so-called “army of indexers” (p. 110). This concept is the theoretical foundation of what would become the Science Citation Index and ultimately the Web of Science.

In 1960, the Institute for Scientific Information (ISI) came into being after its name was changed from Eugene Garfield Associates Inc. ISI was later acquired by Thomson Reuters, and was eventually merged into Clarivate Analytics. In 1964, Eugene Garfield created the first regular quarterly, print edition of the Science Citation Index (SCI) (Cawkell and Garfield 2001; Lazerow 1974), which was followed by the Social Science Citation Index (SSCI) and the Arts and Humanities Citation Index (A&HCI) in 1973 and 1978, respectively (Klein and Chiang 2004). These indices include only journals that are deemed to be of high quality and strong impact. As of November 2, 2017, the three indexes cover 8927, 3272, and 1787 journals, correspondingly. Although some of the data in these indices had been available since the 1970s through other systems such as Dialog, it was not until 1997 when ISI, by then a part of the Thomson Corporation, merged this data into an online interface called the Web of Science (Clarivate Analytics 2017; Meho 2007).

Today, Clarivate Analytics’s WoS has evolved into one of the world’s premier scientific citation search, discovery, and analytical information platforms. It is used as both an academic library research tool as well as a rich dataset for large-scale data-intensive studies across myriad academic fields. WoS contains tens of millions of bibliographic records comprising billions of citation connections and additional metadata fields; and many thousands of additional items are ingested on a daily basis. The WoS platform also includes software productivity functionality including EndNote and InCites (Clarivate Analytics 2017).

Another core component of the Web of Science is the Journal Impact Factor (JIF). As is well documented elsewhere, the “impact factor” is the measure adopted in the InCites Journal Citation Reports (JCR) for SCI and SSCI (Garfield 1977, 2007; Meho 2007). By calculating the number of citations received by all the papers published during a rolling 2-year window (Garfield 1972, 1996), it aims to evaluate the relative importance of scientific journals. Despite its popularity, the method for calculating the impact factor is a subject of on-going deliberation causing some researchers to feel that the JIF is not a consistently reliable indicator of research quality (Amin and Mabe 2004; Cameron 2005; Coelho et al. 2003; da Silva and Bernès 2017; Hansson 1995; Kumar et al. 2009; Seglen 1997; Simons 2008). On the other hand, the on-going discussions, writing, and broad debate around the value of the impact factor and WoS also suggest not only the significance of the impact factor in contemporary scientific evaluation, but also the prominent role played by the Web of Science and its related products.

Throughout his career, Garfield deftly balanced the roles of entrepreneurial businessman, imaginative academic researcher, and thoughtful mentor. However, and rather curiously, his commercial contributions have rarely been examined from the perspective of scientometrics, a field that has been significantly advanced both by Garfield’s research and for-profit products. His colleagues and disciples from ISI, the Thomson Corporation, Thomson Reuters, and Clarivate Analytics have consistently followed his lead with published applied research work based on the Web of Science platform and citation dataset. Perhaps most notable is Henry Small and his codification of co-citation analysis in the early 1970s (Small 1973), although over the past few decades, others have added to this body of accomplished scientometric analytical work (e.g., Pendlebury 1993; Ponomarev et al. 2014; Pringle 2008; Shuai et al. 2017; Zhang et al. 2017). This research both contributes new insights to bibliometric academic knowledge and also informs on-going product innovation for the Web of Science platform and toolset; some of this research is included in our analysis in this paper.

The concept of scientometrics was first coined by Nalimov and Mulchenko (1969) to denote “all aspects of the literature of science and technology” (Hood and Wilson 2001, p. 293). Since then, the term has been gradually refined and is now generally accepted to mean the quantitative aspects of the studies of science and technology (Sengupta 1992; Van Raan 1997), which has significant overlap with the concept of bibliometrics (Broadus 1987). Zupic and Čater (2015) identified five major methods used in bibliometric studies, including citation, co-citation, bibliographic coupling, co-author, and co-word, the first three of which can be applied on multiple levels of entities. All these methods, from different angles, deal with the quantitative impact of a work or a collection of works, the intellectual and distributive structure of a knowledge domain or a research community, and the relationship between entities (e.g., author, journal, country, etc.) in the space of scientific publication.

Traditionally, scientometric studies are based on the close evaluation of explicit citation connections between scientific documents. During the past decade, as the quantity of research output has risen precipitously and digital data objects have become more important for scientific research and scientists, datasets have also started to become direct research objects in scientometric studies. Under this line of scholarship, researchers have traced the quantitative scientific impact of specific datasets (Apai et al. 2010; Belter 2014; He and Han 2017; He and Nahar 2016; Mayo et al. 2016; Peters et al. 2015, 2016). A related topic that has recently attracted substantial interest is the quantification of the impact on original papers, typically measured in increased citations, after a paper’s dataset has been made openly available (e.g., Dorch 2012; Gleditsch et al. 2003; Henneken and Accomazzi 2011; Ioannidis et al. 2009; Pienta et al. 2010; Piwowar et al. 2007; Piwowar and Vision 2013). In our view, these findings all support the growing importance of research datasets and suggest their emerging value as objects of focus for scientometric studies.

Moreover, a few studies have investigated the different patterns of digital object mentions and citations across disciplinary boundaries. For example, Chao (2011) found that earth science datasets are primarily cited in physical science and interdisciplinary journals. More recently, Zhao et al. (n.d.) identified highly variant patterns of the ways datasets are mentioned, cited, and described in scientific papers across 12 disciplines. Both of these studies suggest that academic field of study is a key variable of how data objects are addressed in scientific publications. One approach to scientometric studies of digital objects is the use of automatic natural language processing (NLP) techniques to investigate the grammatical patterns of a large body of texts. NLP methods, especially part-of-speech (POS) tagging, sentiment analysis, and name-entity recognition, have been increasingly used by scientometricians to answer a wide range of research questions (Demarest and Sugimoto 2015; Pan et al. 2015; Small 2011; Teufel et al. 2006). Following the tradition of citation content and context analysis established by Henry Small (Small 1982), these methods have deepened our appreciation of the impact of individual documents or objects, by taking the citation or mention context into consideration.

Despite the prominent position held by the Web of Science database, and its associated products and features, in scientific studies across different knowledge domains, the WoS platform and dataset have been examined only minimally using the theories and methods that they have supported. To better understand Eugene Garfield’s contributions and to extend the scholarship of scientometric examination of data objects, this paper aims at investigating how the Web of Science database is mentioned in published scientific papers. More specifically, we will answer the following questions:

- How have Web of Science and its products been mentioned in scientific literature?
- How are the papers that mention Web of Science distributed across different document genres, institutions, countries, and knowledge domains?
- How have these distribution patterns changed over time?
- What additional words are used most frequently along with mentions of WoS and its components in the abstracts of papers?

We believe that the answers to these questions will help to illustrate some of the depth and breadth of the impact of WoS as both a search tool and a bibliographic dataset over time and across academic fields.

## Methods

Data was collected in the web interface of WoS during November 14–15, 2017. Four terms related to WoS, “web of science,” “impact factor,” “science citation index,” and “journal citation report,” were used to search in the Web of Science Core Collection; this includes academic materials published in more than 18,000 scholarly journals^2^. We choose these four terms for our search criteria based on our literature review and our intuition and general experience with scientometrics and the WoS platform. Besides the query terms, we also limited our data to only include research and review articles written in English and published between 1997 and 2017. We chose this time period as it is contiguous with the existence of the Web of Science itself, which debuted in 1997. We found 19,478 papers meeting our criteria, and downloaded their metadata records for our analysis. Unless otherwise specifically noted in the following sections of this paper, when we refer to “Web of Science,” all the results connected to these terms are included.

We focused our analysis on the following aspects of the metadata records offered by WoS: journals, subject categories, institutions and countries of all authors. These metadata fields are significant to the present study because they are the strongest indicators of the impact of WoS in some space, either geographical or intellectual. To understand the knowledge domain of each article, we extracted the InCites Journal Citation Reports (JCR) Citation Index categories from the “WC” field in the downloaded dataset. We then mapped all these categories into Essential Science Indicator (ESI) classes using an approach similar to that reported by Yan (2014). ESI has 22 classes, compared to 252 in JCR. By having a much smaller number of categories, the ESI scheme can thus reflect a broader view of all knowledge domains. It is also worth noting that only science and social science domains are included in the original ESI schema. We added new categories, such as “Arts & Humanities,” to those in ESI to more comprehensively cover the scope of our WoS dataset.

For the geographical information associated with each paper, we relied upon the country information from the “C1” field of the downloaded dataset, even though country and institution are not mentioned in the address statement in every bibliographic record. We applied text mining techniques to extract country and institution names from the address statements. All descriptive data was analyzed and visualized using the software R (R Core Team 2016).

To investigate the contexts in which WoS is mentioned in the papers, we analyzed the words that are grammatically connected to the WoS entities in the abstracts of all the sampled papers. To this end, we parse the *dependency networks* (Carroll et al. 1999; Klein and Manning 2003) of all the sentences in the abstract, and analyzed only the verbs and nouns that are directly dependent with the phrases that are related to WoS. We tagged all the WoS-related phrases in advance, to avoid the phrases being parsed as individual words (for example, we changed “Web of Science” into “WebOfScience,” and “Institute for Scientific Information” into “InstituteForScientificInformation” in our data.) We used the Stanford CoreNLP software (Manning et al. 2014) as implemented in the “coreNLP” package of R (Arnold and Tilton 2016) to conduct this analysis.

## Results

### General distributive patterns of all papers

**Fig. 1:**
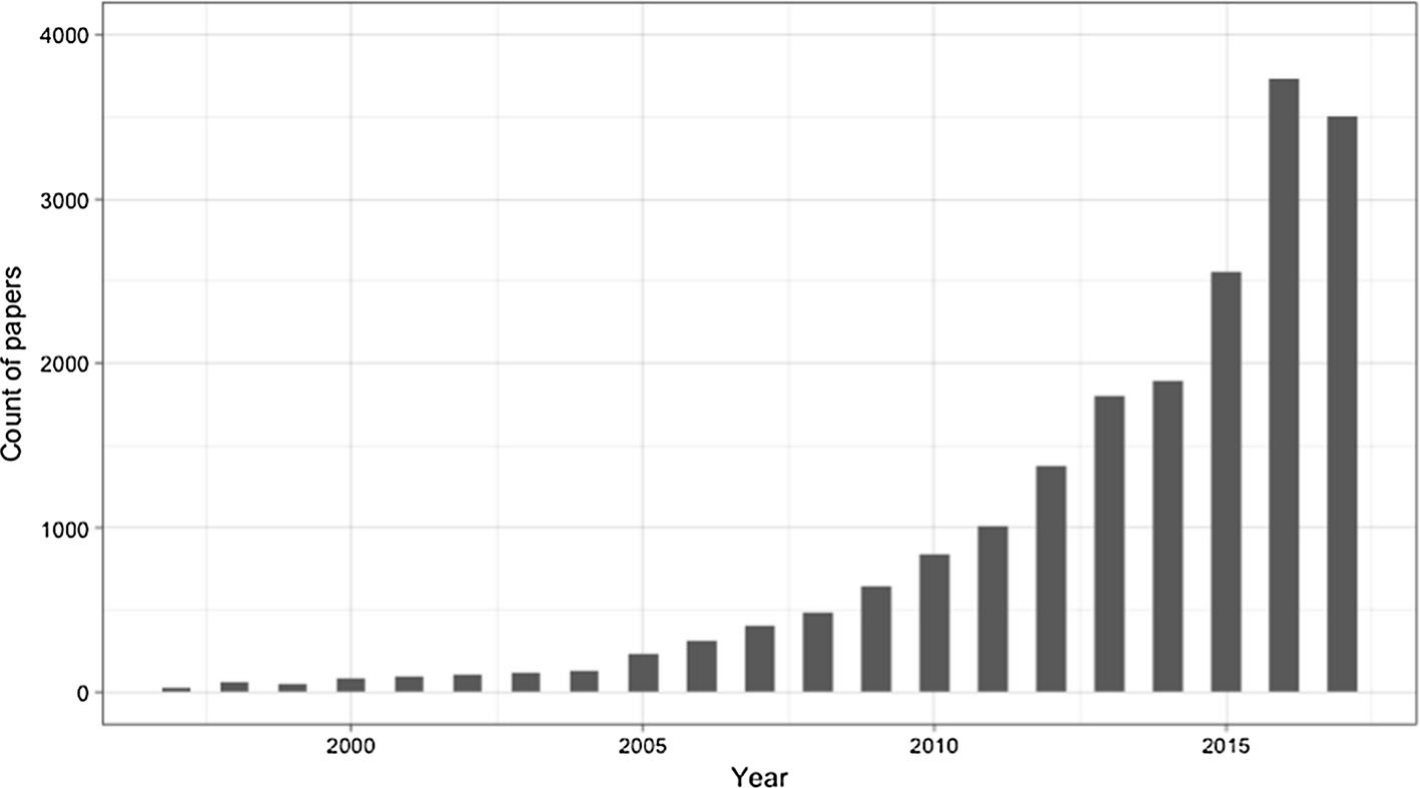
Yearly distribution of all papers mentioning Web of Science

As shown in Fig. 1, there has been a dramatic and steady growth in the number of papers mentioning WoS during the past 20 years. We identified 3739 papers that mentioned any WoS-related concept published in 2016, more than 120 times the size of papers published in 1997, and about 0.21% of all papers published that year. WoS debuted as an online product in 1997 so, given the eventual pervasiveness of the tool, it seems intuitive that there would be some increase in its use over time. Regardless, we see this growth as a solid indication of the important role played by WoS in the overall academic community.

**Fig. 2:**
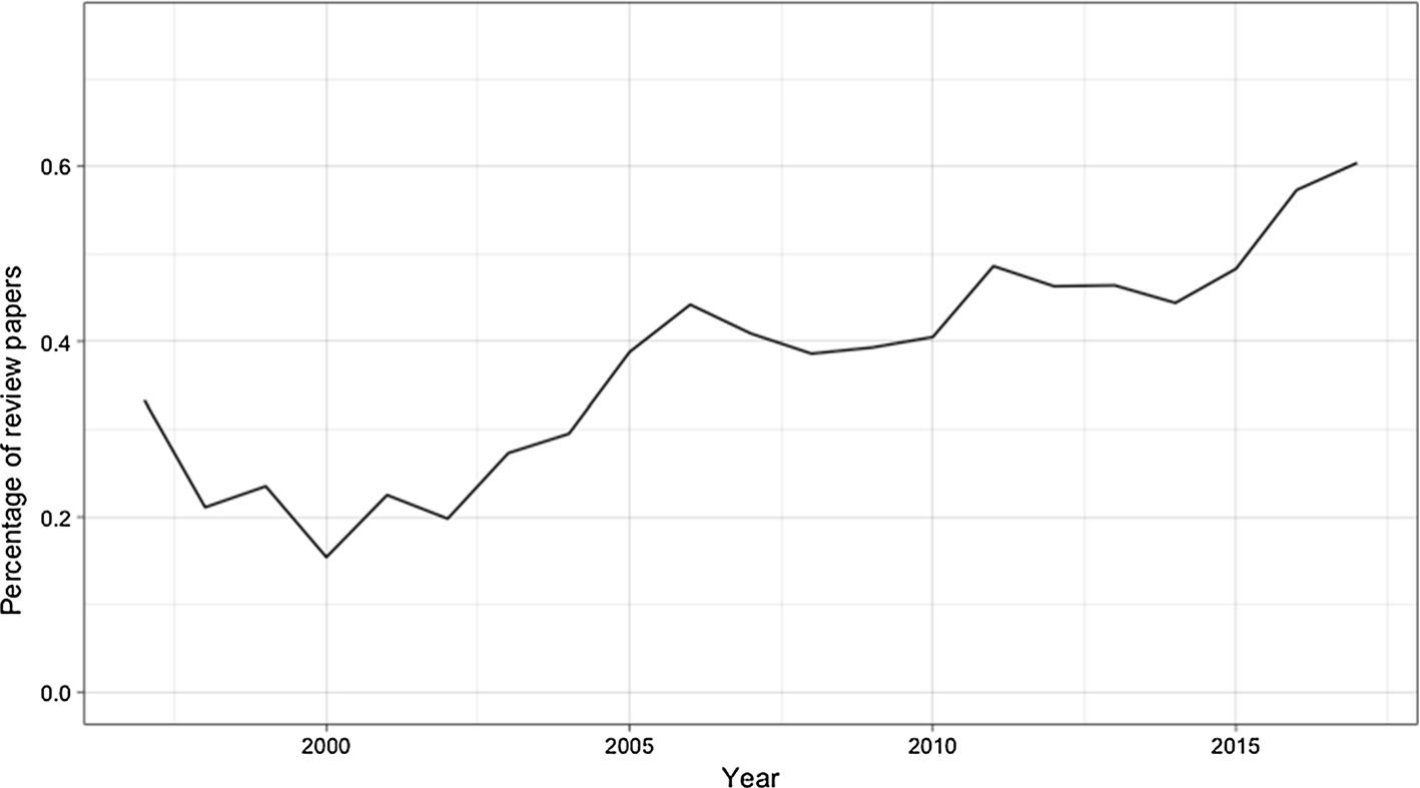
Percentage of review papers mentioning Web of Science

We are specifically interested in how WoS has been mentioned in review papers versus research papers. As shown in Fig. 2, overall, the percentage of review papers in our dataset has been increasing since the beginning of the twenty-first century: after 2015, more than half of all papers published every year are review papers. This highlights the importance of WoS data and tools not only as the instrument for empirical scientific studies, but also for studies to resolve the differences between a group of reports or to find new research areas based on existing efforts.

There are 3905 unique journals included in our dataset; of these, 10 journals cover 4232 papers in the sample (or 21.7% of all papers). Among these journals, *Cochrane Database of Systematic Reviews (CDSR)* (1359), *PLoS ONE* (766), and *Scientometrics* (757) are the three top journals in the list. All the other journals published fewer than 250 papers mentioning WoS-related entities. Most of the journals in the top 10 list belong to medical science (such as *Medicine*, *Oncotarget* and *International Journal of Clinical and Experimental Medicine*) and information science (such as *Journal of Informetrics* and *Journal of the American Society for Information Science and Technology*). We also split all papers into four groups by the publication year (1997–2002, 2003–2007, 2008–2012, and 2013–2017), and investigated the top journals by each group. One of the most salient patterns in this table is that journals in the field of library and information science have been gradually replaced by journals in other fields, especially medical science: five of the top 10 journals in the first period are from information science verses only two in the last period.

### Distribution of papers by country and institution

Table 1 displays the top 10 countries that are connected to all authors in the sampled papers. These countries contribute to 15,656 papers in our dataset (or 80.3% of all papers). It is worth mentioning that this table is relatively consistent with other country-level rankings based on a large quantity of academic publication data, such as the Nature INDEX^3^.

**Table 1:** Top 10 country of origin of all authors

Country	Count
China	5096
USA	4076
England	2614
Canada	1321
Australia	1290
Netherlands	1068
Italy	907
Germany	851
Brazil	799
Spain	734

Table 2 displays the frequencies of country of origin of first authors for papers in our dataset. Although this list shares all the same countries as Table 1, the order of the countries varies. For example, China decreases from No. 1 in the previous table to No. 3 in this one.

**Table 2:** Top 10 first author country of origin

Country	Count
USA	1951
England	998
China	904
Canada	492
Australia	473
Netherlands	436
Brazil	337
Spain	299
Italy	284
Germany	269

**Fig. 3:**
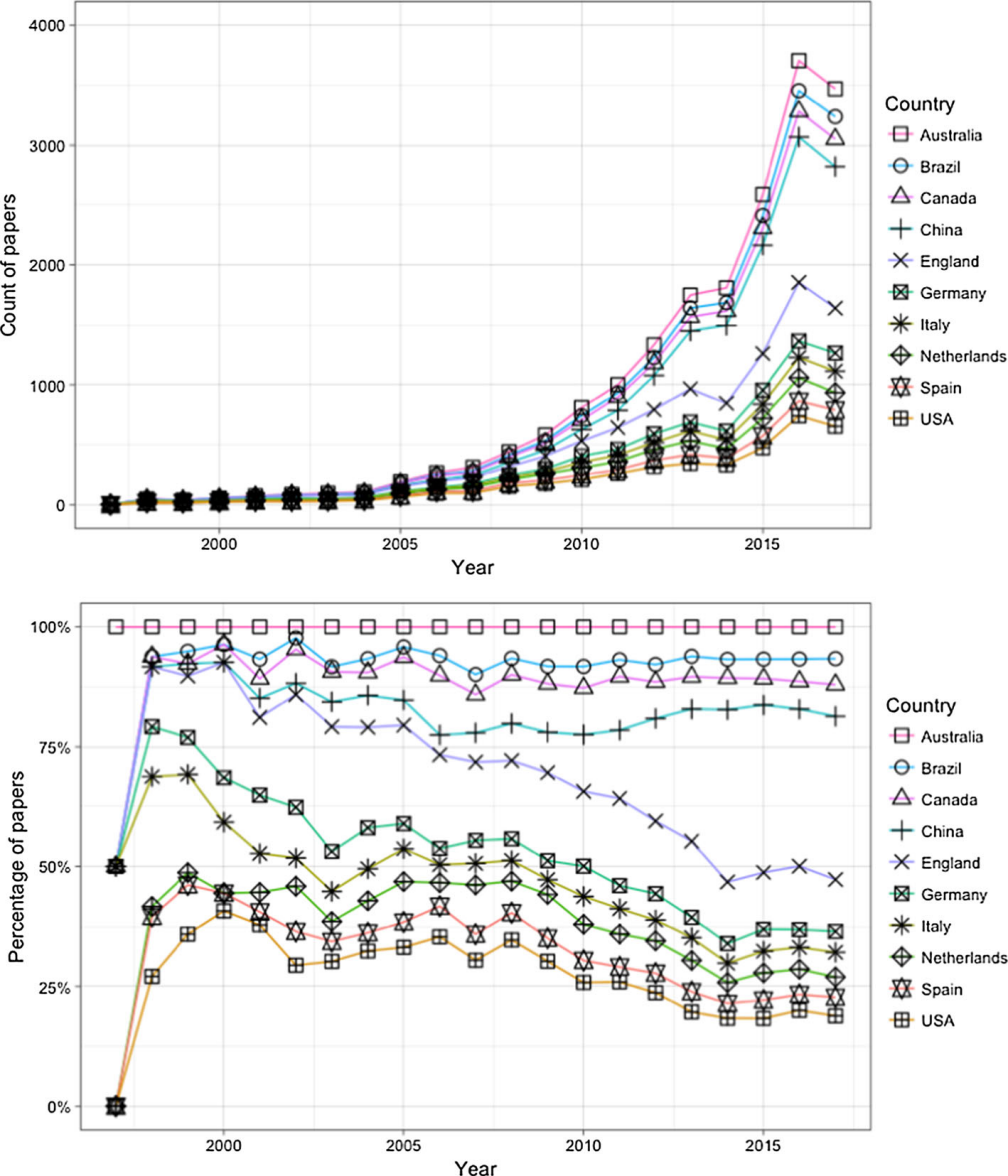
Number and percentage of papers by Top 10 countries based on all authors by year

**Fig. 4:**
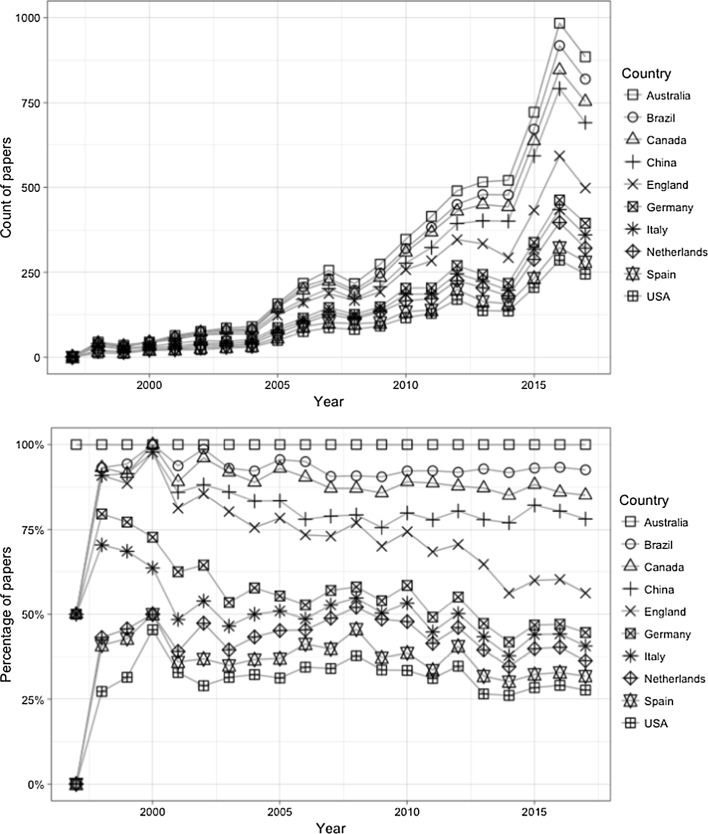
Number and percentage of papers by Top 10 countries based on first authors by year

Figure 3 displays the number of papers produced in the top 10 countries by year, as well as their relative sizes within all the papers published by the top 10 countries (for all the figures in this paper, the size of the category is represented by the space under the line). It shows a similar increasing pattern of the number of publications among these countries that is similar to the whole sample. Notably, the rapid growth of China can also be observed easily: it surpassed the USA as the most productive country in 2013. Figure 4 shows the absolute and relative sizes of the top 10 countries with only the first authors counted.

Besides the country of all authors, we also analyzed the top institutions from our dataset. University of Toronto (527), Mayo Clinic (483), and Sichuan University (470) are the three most frequently occurring institutions from 1997 to 2017. Two other institutions (China Medical University and Zhejiang University) have also published than 400 papers each. Following the example of Table 3, the top institutions by year group are displayed in Table 4. Mirroring the patterns we observed with countries, the number of papers connected to institutions outside America and Europe, especially China, has grown substantially during the past 20 years: all but three of the top institutions in the last group are from China.

**Table 3:** Top 10 frequently occurring academic journals from the periods of 1997-2002, 2003-2007, 2008-2012, and 2013-2017

Journal	Count	Journal	Count
Scientometrics	40	Cochrane Database of Systematic Reviews	162
Haematologica	36	Scientometrics	100
Journal of Documentation	11	Journal of the American Society for Information Science and Technology	30
Journal of Information Science	10	JAMA—Journal of the American Medical Association	15
Journal of the American Society for Information Science and Technology	9	Annals of Pharmacotherapy	12
Physical Review D	7	British Medical Journal	12
Annals of Pharmacotherapy	6	Journal of Advanced Nursing	9
Web of Knowledge—A Festschrift in Honor of Eugene Garfield	6	American Journal of Gastroenterology	8
British Medical Journal	5	Evidence Based Library And Information Practice	8
Journal of the American Society for Information Science	5	Journal of Information Science	8
Cochrane Database of Systematic Reviews	493	Cochrane Database of Systematic Reviews	704
Scientometrics	221	PLoS ONE	669
Journal of the American Society for Information Science and Technology	96	Scientometrics	396
PLoS ONE	95	Medicine	245
Journal of Informetrics	66	Oncotarget	234
Health Technology Assessment	43	International Journal of Clinical and Experimental Medicine	225
British Medical Journal	39	BMJ Open	191
Collnet Journal of Scientometrics and Information Management	27	Tumor Biology	119
Breast Cancer Research and Treatment	23	Scientific Reports	112
European Urology	23	Journal of Informetrics	98

**Table 4:** Top 10 most frequently occurring institutions from the periods of 1997–2002, 2003–2007, 2008–2012, and 2013–2017

Institution	Count	Institution	Count
Univ Calif San Francisco	10	Univ Toronto	43
Univ Genoa	10	Univ Alberta	31
Hosp Univ Canarias	9	Univ Amsterdam	29
McMaster Univ	8	Univ Calif San Francisco	27
Univ Birmingham	7	McMaster Univ	23
Johns Hopkins Univ	6	Johns Hopkins Univ	18
Off Naval Res	6	Taipei Med Univ	18
Royal Sch Lib & Informat Sci	6	Tufts Univ	18
Inst Sci Informat	5	Harvard Univ	16
Univ Bologna	5	Leiden Univ	16
Univ Toronto	155	China Med Univ	397
Mayo Clin	143	Sichuan Univ	384
Leiden Univ	99	Zhejiang Univ	376
Univ Alberta	96	Nanjing Med Univ	367
Univ Tehran Med Sd	95	Univ Toronto	328
Harvard Univ	88	Mayo Clin	326
Sichuan Univ	84	Univ Tehran Med Sci	291
McMaster Univ	78	Sun Yat Sen Univ	255
Fudan Univ	76	Fudan Univ	245
Univ Amsterdam	75	Huazhong Univ Sci & Technol	244

### Distribution of papers by scientific field

Our dataset covers 232 of all the 252 JCR subject categories used in the Web of Science Core Collection. Table 5 shows the top 10 subject categories covered by all the papers. Moreover, we did extra queries in WoS database using each of these 10 subject categories combined with other parameters described in the section of data collection (English academic and review papers published between 1997 and 2017). Based on the results, we calculated the percentage of papers mentioning WoS entities in the total number of papers under each category. Not surprisingly, *Information Science & Library Science* has the highest percentage of papers mentioning WoS, suggesting the importance of the database and tools in this field. *Medicine, General & Internal* also has a significantly higher percentage than the rest of the top categories.

**Table 5:** Top 10 JCR subject categories

Subject	Count	Percentage of WoS papers
Medicine, General and Internal	2735	0.0078
Information Science & Library Science	1896	0.0295
Oncology	1286	0.0022
Multidisciplinary Sciences	1068	0.0021
Computer Science, Interdisciplinary Applications	1000	0.0049
Surgery	970	0.0014
Public, Environmental & Occupational Health	937	0.0025
Pharmacology & Pharmacy	783	0.0013
Clinical Neurology	684	0.0017
Medicine, Research & Experimental	601	0.0019

From both Tables 1 and 5, it is not difficult to observe that most of the papers we retrieved belong to medical science. This observation is supported as JCR subject categories were mapped to ESI classes. Table 6 lists the top 10 ESI classes covered by all papers, where *Clinical Medicine* is the dominant knowledge domain in our data.

**Table 6:** Top 10 ESI categories

Field	Count
Clinical Medicine	11,909
Social Sciences, general	3486
Computer Science	1482
Science, Multidisciplinary	1068
Biology & Biochemistry	935
Psychiatry/Psychology	862
Pharmacology & Toxicology	842
Molecular Biology & Genetics	577
Engineering	462
Environment/Ecology	454

**Fig. 5:**
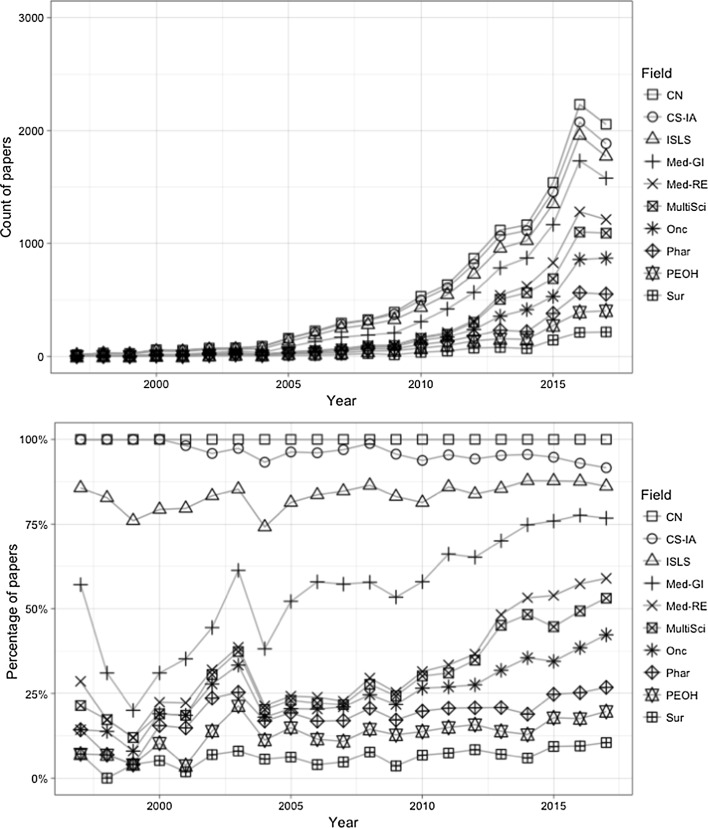
Top 10 JCR subject categories by year (CN: Clinical Neurology; CS-IA: Computer Science, Interdisciplinary Applications; ISLS: Information Science & Library Science; Med-GI: Medicine, General & Internal; Med-RE: Medicine, Research & Experimental; MultiSci: Multidisciplinary Sciences; Phar: Pharmacology & Pharmacy; Sur: Surgery; Onc: Oncology; PEOH: Public, Environmental & Occupational Health)

**Fig. 6:**
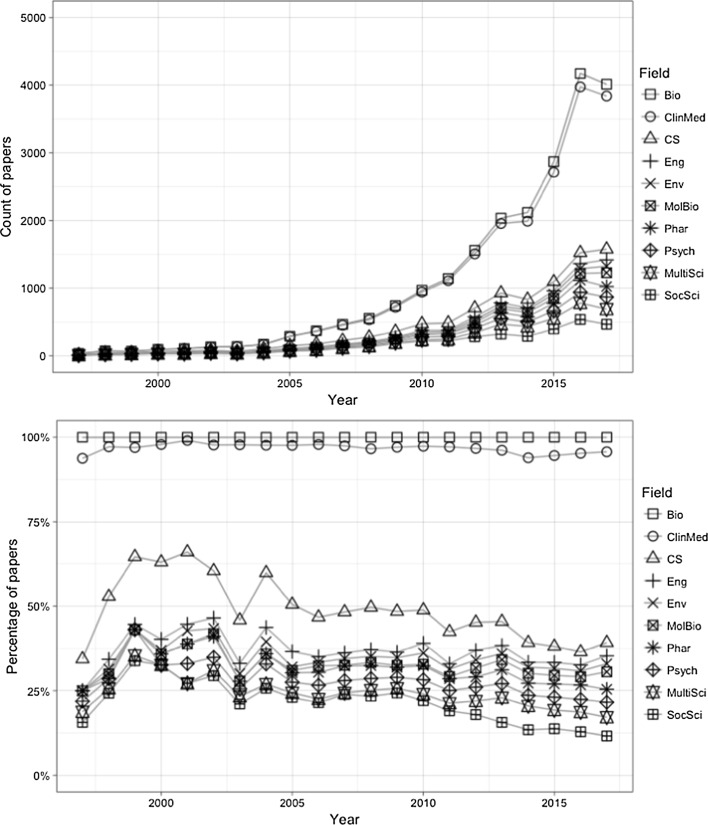
Top 10 ESI subject categories by year (Bio: Biology and Biochemistry; ClinMed: Clinical Medicine; CS: Computer Science; Eng: Engineering; Env: Environment and Ecology; MolBio: Molecular Biology and Genetics; Phar: Pharmacology and Toxicology; Psych: Psychiatry and Psychology; MultiSci: Science, Multidisciplinary; SocSci: Social Science, general)

We plotted both classification schemes on the timeline, as displayed in Figs. 5 and 6. Based on Fig. 5, *Information Science & Library Science* is the field where WoS was mentioned the most before 2005. After 2005, it was gradually surpassed by other fields such as *Medicine, General & Internal* and *Oncology*. Most of the top categories in this graph had a substantial growth during the past decade.

As for the ESI subject classes, *Clinical Medicine* has been the dominant domain during most of the twenty-first century. All the other categories, except for *Social Science*, fail to distinguish themselves from others, despite their steady growth in terms of the total number of papers after 2005.

### Textual context of Web of Science mentions

In this section, we present only the results of NLP analysis for the term “Web of Science.” This choice was made for two reasons: first, of all the contextual terms identified in this analysis, 71.7% (27,764 out of 38,641 words) are connected to “Web of Science;” second, “Web of Science” is also the broadest term that represents the overall topic of this paper. In total, we found 5231 verbs and 15,853 nouns grammatically connected to “Web of Science” from all the abstracts we examined.

Table 7 displays the five verbs that are the most frequently used, that are grammatically connected to “Web of Science.” All these verbs are strongly connected to the context in which the authors use WoS as a data source, and the use is described in the method statement. This connection is reflected in both the types of grammatical connection between the verbs and “Web of Science” and the quotes we have examined. For example, for the verb “search,” its dependency relationship with “Web of Science” falls into the following three categories of Universal Dependencies (Nivre et al. 2016) in more than 93% (1876 of 2000) cases:

**Table 7:** Top five most frequently occurring verbs linked to “Web of Science”

Verb	Count
Search	2000
Use	723
Conduct	333
Perform	310
Identify	176

- “dobj” (the noun phrase is the direct object of the verb)
- “nsubjpass” (the noun phrase is the syntactic subject of a passive clause)
- “nmod” (a word is nominal dependent of another noun or noun phrases, as an attribute or complement)

A representative quotation for each category is listed below (emphasis added):

Relevant studies were identified by **searching** PubMed, EMBASE, and ISI **Web of Science** for articles published before April 2017. (Wang et al. 2017, p. 59666)

PubMed, Embase, and **Web of Science** for publications were **searched** using the keywords of miR-150 and human cancer. (J. Yan et al. 2017, p. 1187)

We **searched** in EBSCO, PsycINFO, Google Scholar, **Web of Science**, and NCBI databases and other articles manually from lists of references of extracted articles. (Piqueras, Martín-Vivar, Sandin, San Luis, & Pineda 2017, p. 153)

The same pattern was found for the four other top verbs listed in Table 7. These verbs share a similar semantic meaning with “search” (as a method of data collection), and the majority instances of their relationship with “Web of Science” fall into the aforementioned categories.

Even though building a fuller typology of the context of mentioning WoS is beyond the scope of this paper, we identified three different contexts distinct from those discussed above. These contexts include Web of Science as mentioned to set the stage for the research, Web of Science as described in the result statement, and Web of Science as mentioned in the conclusion statement. An example of each category is offered below (with emphasis added):

An archetype of these databases **is** the **Web of Science** (WoS) that stores scientific publications. (Orosz, Farkas, & Pollner 2016, p. 829)

In the **Web of Science published** by the Institute for Scientific Information, the earliest literature record was in April, 1995. (Leng, He, Li, Wang, & Cao 2013, p. 1286)

Apparently, the ISI **WoS is** more suitable to compare the research productivity of different countries, authors or institutions. (Jelercic, Lingard, Spiegel, Pichlhöfer, & Maier, 2010, p. 582)

In all these papers, these contexts are not mutual-exclusive: sentences serving different purposes coexist in all these three papers. Moreover, the dependency patterns of these three sentences are also shared by the sentences under the first scenario. For example, many cases of “be” and “publish” as the contextual verbs of Web of Science are also used to introduce Web of Science as the data source.

We also analyzed the most frequent nouns directly linked to Web of Science mentions. Table 8 shows the top 10 nouns identified from our sample.

**Table 8:** Top 10 most frequently occurring nouns linked to “Web of Science”

Noun	Count
Database	2489
Medline	2298
Embase	1652
Search	805
Scopus	799
Library	644
CINAHL	389
Scholar	368
Register	298
PsycINFO	253

Overall, nouns were more challenging to parse and analyze properly due to the nature of the specific words most frequently associated with WoS mentions. For example, seven of the 10 nouns included in this list are proper nouns; they are all product names of other databases (such as “Medline,” “Embase,” “Scopus,” “CINAHL,” and “PsycINFO”) that are listed together with Web of Science as the search tool or data source for a particular study. Some of these names, especially “Scopus” and “PubMed” (the latter name fails to make this list but is still frequently mentioned), are sometimes mistakenly identified as verbs by the parser, which reduces their presence in this list. In some other cases, these terms are a part of the name of a database; examples of this category include “library” (“Cochrane Library”), “scholar” (“Google Scholar”), and “register” (“Cochrane Central Register of Controlled Trials”). This situation is because we did not preprocess the names of other databases. “Database” and “search” are the only two words in the list that are primarily used as regular nouns. Regardless, we feel that our analysis of the proper nouns closely linked with Web of Science add a new dimension to the context of using Web of Science as a data source, that it is also frequently used in combination with other databases. This line of inquiry is also an area where further research could likely uncover additional insights.

## Conclusions

This paper, for the first time, offers a selective review of the impact of WoS as a research object from the perspectives of scientometrics and NLP. Our initial presumption, based on our literature review and personal experience, was that WoS held a notable position as a research tool and dataset across many academic fields and a close analysis would enable us to quantify this. We first measured its scientific impact in terms of the total number of papers in the Web of Science databases, and then analyzed the distributions of all the sampled papers on the levels of journal, country, institution, and knowledge domain, with or without the publication year considered. Moreover, we conducted an exploratory NLP analysis to extract the verbs and nouns as the direct context of mentioning Web of Science in the abstract of all the papers. We identified the most frequent words and their linguistic connections to “Web of Science,” and discussed what these patterns might suggest about the use and mention of Web of Science in the scientific texts.

Our descriptive analysis using scientometric techniques supports the fast-growing impact of Web of Science based on scientific publication: the number of papers mentioning Web of Science has risen from 30 in 1997 to more than 3700 in 2016, and the percentage of papers mentioning Web of Science in all papers has also been increasing every year.

More importantly than its sheer count, Web of Science is also heavily used by global researchers in nearly every knowledge domain. Based on incomplete address information, we identified authors from 125 countries all over the world. Our sample also covers 232 out of the 252 Web of Science subject categories. Among all these fields, *Library and information science* is the category with the most papers and still has the highest percentage of papers mentioning Web of Science. However, many other fields, especially those in medical science, have surpassed *Library and information science* in the productivity of using Web of Science data or at least mentioning its name. The knowledge domain of *Clinical Medicine*, based on the ESI classification scheme, is the dominant domain identified in this analysis, with more than three times as many papers as the second largest domain, *Social Science, general*.

To enhance our insights of Web of Science based on quantitative measures, we also adopted NLP techniques to dig deeper into the contexts in which Web of Science is mentioned in the abstract of our sampled papers. By just focusing on verb and nouns that are directly dependent with Web of Science, we concluded that the most important reason researchers mention WoS is that it is used as a source of data, often in combination with other databases. This conclusion was drawn based on the meanings and linguistic patterns of the most often occurring verbs and nouns. We also found that there are other types of contexts in which WoS is documented in the introduction, results, and conclusion statements in the abstracts. Even though this scheme per se is not the aim of this paper, the four categories are consistent with findings of many researchers in other linguistic analysis of academic abstracts, that an ideal abstract is supposed to cover contents from all of the introduction, method, result, and conclusion sections (e.g., Salager-Meyer 1990, 1992; Samraj 2005; Swales 1981). Based on these studies, our results also suggest that WoS, as a data object, could serve multiple functions within scientific texts, besides being used as a data source. And these different contexts could be accompanied by distinguishable language patterns in scientific texts that can be automatically identified by NLP algorithms. We are hoping to conduct future studies that more systematically address this very important question between scientometrics and NLP.

Most importantly, we are confident that this study helps to quantify the general significance of the Web of Science over the past 20 years. All of the findings of this paper demonstrate that WoS is an increasingly significant scientific instrument across countries and knowledge domains, being used by global scientists in different ways to answer scientific questions. This is a tremendous intellectual debt the scientific community owes to Eugene Garfield.

## Limitations

While we made every practical effort to be thorough and comprehensive in our data collection and analysis, we recognize there are a few limitations to the current study as follows:

- We only used metadata from WoS. Use of metadata or full text from additional sources could potentially yield different results or could be an avenue for further research to complement this study.
- We focused on “review” and “article” document types thus excluding things like opinion pieces and letters to the editor that may also include substantive references to WoS and related entities.
- We analyzed only English language scholarly material even though WoS also indexes material originally published in other languages.
- As noted in “Textual context of Web of Science mentions” section, the development of a fuller typology of words related to WoS mentions was outside the scope of this study but could likely be considered for future work.
- Also noted in the Conclusion just above, there are probable distinguishable language patterns for different standard sections of scientific papers (abstracts, results, etc.) that could be systematically identified and analyzed.

We are confident that these limitations are reasonable considering the scope of this current study but also feel future research may benefit from expanding this work to encompass some of the items listed above.

## Footnotes

1 All his works are available at his ResearcherID page at: http://www.researcherid.com/rid/A-1009-2008.

2
https://clarivate.com/products/web-of-science/web-science-form/web-science-core-collection/.

3
https://www.natureindex.com/annual-tables/2016/country/all.
